# After-hours work-related technology use, work-to-family interference, and exhaustion among Korean wage workers: gender patterns in associations and covariate-standardized levels

**DOI:** 10.3389/fpubh.2026.1939418

**Published:** 2026-08-21

**Authors:** Hwanjin Park, Kounseok Lee

**Affiliations:** 1 Department of Occupational and Environmental Medicine, Kangbuk Samsung Hospital, Sungkyunkwan University School of Medicine, Seoul, Republic of Korea; 2 Department of Psychiatry, College of Medicine, Hanyang University, Seoul, Republic of Korea; 3 Department of Psychiatry, Schulich School of Medicine and Dentistry, Western University, London, ON, Canada

**Keywords:** after-hours connectivity, exhaustion, gender, occupational mental health, survey weighting, work-to-family interference

## Abstract

**Introduction:**

After-hours work-related technology use may erode boundaries between work and private life. We examined whether after-hours technology use was associated with exhaustion through work-to-family interference (WIF), whether the modeled associations differed by gender, and whether men and women had different covariate-standardized outcome levels.

**Methods:**

Pooled cross-sectional data were obtained from 56,640 wage workers in the 6th (2020–2021) and 7th (2023) Korean Working Conditions Survey. Primary analyses used wave-specific standardized survey weights, probability-weighted linear regression with HC3 robust standard errors, and 5,000 multiplier-bootstrap replications.

**Results:**

After-hours technology use was associated with higher WIF (B = 0.064, 95% CI 0.053–0.074; standardized beta = 0.10) and exhaustion in the total-association model (B = 0.042, 95% CI 0.031–0.052; beta = 0.07). WIF was associated with exhaustion (B = 0.329, 95% CI 0.314–0.344; beta = 0.34), and the remaining direct association was small (B = 0.021; beta = 0.03). The technology-by-gender interactions in the WIF and total-association models and the gender difference in the conditional indirect association met the stated post hoc equivalence criteria; the other path interactions were not statistically significant. Indirect associations were 0.021 (95% CI 0.017–0.025) for men and 0.018 (95% CI 0.014–0.021) for women; the difference was −0.003 (95% CI −0.008 to 0.002). After standardization to a common distribution of measured paid-work characteristics, women had higher exhaustion than men (difference = 0.087, 95% CI 0.066–0.109). Exploratory categorical analyses suggested a localized, non-monotonic gender difference at several-times-per-month use; this pattern was observed in both waves without detectable wave heterogeneity.

**Discussion:**

The associations were small in absolute magnitude, and the cross-sectional design precludes causal interpretation.

## 1 Introduction

The rapid expansion of work-related technology use beyond standard working hours has fundamentally reshaped the boundaries between work and private life. Digital communication tools such as smartphones, email, and messaging platforms allow work to extend into evenings, weekends, and domestic spaces, rendering temporal and spatial boundaries increasingly permeable. Scholars interpret this shift not simply as a technological change, but as a transformation in the organization of work, in which availability and responsiveness beyond formal working hours have become increasingly institutionalized (1). This development matters for public health because psychosocial working conditions are important social determinants of population mental health (2), and constant connectivity now reaches large segments of the workforce. A growing body of evidence indicates that after-hours connectivity reduces opportunities for recovery, weakens the distinction between work and non-work domains, and is associated with elevated strain, work–family conflict, and diminished wellbeing (3–5).

These concerns are particularly salient in the Republic of Korea, where long working hours coexist with widespread smartphone use and messenger-based work communication. Analyses of the Korean Working Conditions Survey (KWCS) have linked after-hours use of work-related communication devices to work–family conflict (6), anxiety symptoms (7), work-related headaches and eyestrain (8), psychological distress following sudden work recall during off-hours (9), sleep disturbance (10), and presenteeism associated with mobile work during non-work hours (11). Reflecting these concerns, a right to disconnect has been legislated in France and a growing number of jurisdictions, and related bills have been discussed in Korea since 2016 without being enacted (8, 12). The right-to-disconnect debate has also attracted scholarly and policy attention elsewhere in East Asia, including Japan, although formal protections remain uneven across jurisdictions (12). Evidence on the associations between after-hours connectivity and worker wellbeing, and on whether these associations vary across social groups, is therefore relevant to occupational health and safety policy. Prior KWCS studies have generally examined work–family conflict or specific health outcomes separately. The present study extends this literature by adding the seventh survey wave, testing exhaustion through a directional work-to-family pathway, evaluating gender moderation across all modeled paths, and formally distinguishing differences in association strength from covariate-standardized outcome levels.

Understanding these dynamics requires a conceptual lens that captures how individuals navigate the boundaries between work and family domains. Boundary theory and work/family border theory conceptualize work–family relations as ongoing processes through which individuals negotiate domains that differ in norms, expectations, and demands (13). From this perspective, after-hours work-related technology use functions as a mechanism that increases boundary permeability and facilitates frequent, often unplanned, role transitions (14). When individuals integrate boundaries excessively, they may experience increased role conflict and struggle to disengage from work (15). Complementing this view, resource-based frameworks—conservation of resources theory, the job demands–resources model, and the work–home resources model—explain how sustained or unpredictable work demands deplete finite personal resources, including time, energy, and attention (16–19). In particular, individuals who fail to achieve psychological detachment from work during non-work time experience fatigue, emotional exhaustion, and broader health consequences (20–23).

Empirical research consistently documents associations between after-hours work-related technology use, work–family conflict (24, 25), and adverse health outcomes (3, 26, 27). Technology-assisted after-hours work has been associated with greater interference between work and family roles, reduced psychological detachment, fatigue, burnout, and sleep disturbance (28–31). These findings suggest that the relevance of after-hours connectivity extends beyond the amount of paid work to sustained cognitive and emotional engagement with work outside formal working hours. After-hours technology use may therefore be understood as a distinct job demand that embeds work within everyday life and alters how workers experience work and family domains. The direction of interference is also conceptually important: work-to-family interference represents the intrusion of work demands into family or personal life, whereas family-to-work interference represents demands moving in the opposite direction (24, 25, 32). Because the exposure examined here originates in the work domain, work-to-family interference provides the most direct primary pathway to exhaustion; family-to-work interference, work-related rumination, and the original five-item composite were examined as sensitivity measures.

An important unresolved question is whether the associations of after-hours connectivity with work–family processes and exhaustion vary between men and women. Some studies suggest stronger work–family conflict among women under conditions of extended availability, often interpreted in relation to their disproportionate involvement in unpaid care and domestic labor (33, 34). However, recent systematic reviews and meta-analyses have not found consistent evidence that gender moderates the relationship between after-hours work-related technology use and work–family conflict (4, 5). It is therefore useful to distinguish two questions that are often conflated: whether the increment associated with greater after-hours connectivity differs by gender, and whether men and women have different adjusted levels of work-to-family interference or exhaustion under a common distribution of measured work characteristics. The latter comparison is conditional on the selected covariates and should not be interpreted as a longitudinal pre-exposure baseline. Feminist organizational scholarship further suggests that group differences in these outcomes may reflect gendered work structures and unequal distributions of paid and unpaid labor rather than differences in individual sensitivity alone (35, 36).

Using pooled data from the 6th and 7th KWCS waves (37), we examined whether after-hours work-related technology use was associated with WIF and exhaustion; whether WIF statistically accounted for part of the technology–exhaustion association; whether gender modified the technology-to-WIF, WIF-to-exhaustion, direct, or total associations; and whether covariate-standardized WIF and exhaustion levels differed between women and men. The weighted linear models constituted the primary analysis. *Post hoc* equivalence analyses contextualized the magnitude of the non-significant linear interactions, and categorical exposure models, wave-specific consistency analyses, alternate work–family measures, expanded covariate sets, and a fully design-adjusted seventh-wave analysis were conducted as secondary or sensitivity analyses.

## 2 Materials and methods

### 2.1 Data and study population

This study used pooled cross-sectional data from the 6th (2020–2021) and 7th (2023) waves of the KWCS, a nationwide survey of employed individuals aged 15 years or older conducted through face-to-face household interviews by the Occupational Safety and Health Research Institute. The KWCS collects information on working conditions, job characteristics, work–life balance, and health-related outcomes, and its sampling design, field procedures, and quality-control procedures are documented elsewhere (37). The survey has been widely used in occupational and social epidemiologic research in Korea (6–8). Pooling the two most recent waves increased the precision of estimates and included observations collected during the pandemic (2020–2021) and post-pandemic (2023) periods. The 6th public release provided a standardized analysis weight, and the 7th provided standardized weights together with stratum and primary sampling unit identifiers. We harmonized the detailed education, occupation, exposure, work–family, exhaustion, and working-time variables before pooling the waves.

To focus on organizationally structured employment and boundary conditions, we restricted the analytic population to wage workers. Self-employed individuals, employers, and unpaid family workers were excluded because they have substantially different authority over schedules, task organization, and boundary permeability, so extended availability does not carry the same meaning for them as for wage workers. The pooled raw sample included 100,733 respondents. We excluded 37,520 non-wage workers, 1,763 respondents without a valid response to the after-hours information and communication technology (ICT) use item, 2,800 without complete responses to all five work–family items, 47 without valid exhaustion data, and 1,963 with missing primary covariates. The common primary sample therefore comprised 56,640 wage workers. The primary WIF models and the five-item-composite sensitivity used this common sample. FIW and rumination sensitivity models used mediator-specific complete cases, with sample sizes reported in Supplementary Table S7. The derivation is shown in Supplementary Figure S1; variable-level missingness by wave and included-vs.-excluded comparisons are reported in Supplementary Table S3.

### 2.2 Measures

#### 2.2.1 After-hours work-related technology use

After-hours work-related technology use was assessed with a single item asking how frequently respondents had used communication devices for work during their free time outside regular working hours in the past month. The item encompasses work-related communication such as receiving messages, checking email, responding to requests, and other task-related contact. Responses were daily, several times per week, several times per month, rarely, or never. The responses were reverse-coded to range from 1 (never) to 5 (daily). The weighted pooled mean was subtracted before forming linear interaction terms. The five response categories were retained without assuming equal spacing in the secondary categorical analyses, and daily vs. less frequent use was examined as an additional sensitivity analysis.

#### 2.2.2 Work-to-family interference and related measures

The primary mediator was work-to-family interference (WIF), calculated as the mean of two identically worded items in both waves: feeling too tired after work to do necessary household work and being prevented by one's job from giving the desired time to family. Responses ranged from always to never and were reverse-coded so that higher scores indicated greater interference. The weighted pooled mean was subtracted before interaction analysis. Family-to-work interference (FIW) was calculated from two items concerning difficulty concentrating on work and insufficient time for work because of family responsibilities. A separate item measured persistent work-related thoughts during non-work time. The original five-item composite—which combined persistent work-related thoughts during non-work time, fatigue that interfered with household responsibilities, insufficient time for family because of work, and interference between work and domestic demands in both directions—was retained as a sensitivity measure. Full item wording and response options appear in Supplementary Table S1.

#### 2.2.3 Exhaustion

Exhaustion was calculated as the mean of two identically administered items addressing physical and emotional depletion related to work: feeling physically exhausted at the end of the working day and feeling emotionally drained by work. Responses were reverse-coded so that higher scores indicated greater exhaustion. The construct is described as exhaustion rather than multidimensional burnout because the two items do not cover all dimensions assessed by comprehensive burnout instruments (38).

#### 2.2.4 Gender and covariates

Gender was analyzed as the binary survey variable provided in the KWCS (0 = men, 1 = women) in order to examine adjusted differences in outcome levels and potential modification of the modeled associations. Primary covariates were age group, education, detailed 10-category Korean Standard Classification of Occupations (KSCO) major group, weekly paid working hours, and survey wave. Age was categorized into six groups, from 15–19 to 60 years or older. Education was harmonized from the detailed seven-level variable as high school or below, associate degree, or bachelor's degree or above. For descriptive presentation only, occupations were summarized as white-collar (KSCO 1–3), service and sales (KSCO 4–5), manual/agricultural (KSCO 6–9), and military (KSCO 10); regression models retained the detailed occupation categories. Weekly paid working hours were treated as a continuous variable and mean-centered, and survey wave was included to account for differences between the two survey periods. Expanded sensitivity models additionally included employment status, industry, shift work, night work, supervisory responsibility, perceived household income adequacy, schedule control, part-time status, and workplace size. Marital status and presence of children were available in the seventh wave and were examined in a wave-specific sensitivity model; comparable harmonized measures of dependent-child age and household composition were not available across both waves.

### 2.3 Statistical analysis

The primary analysis used weighted pooled linear models with the standardized survey weight from each wave normalized to a mean of 1 within wave. HC3 heteroskedasticity-robust standard errors were used because full stratum and primary sampling unit identifiers were unavailable in the public sixth-wave file. The mediator model regressed WIF on mean-centered after-hours ICT use, gender, their interaction, and the primary covariates. The outcome model regressed exhaustion on mean-centered ICT use, mean-centered WIF, gender, ICT-by-gender and WIF-by-gender interactions, and the same covariates. A separate model estimated the total ICT–exhaustion association and its gender interaction. Both unstandardized and standardized coefficients are reported.

Conditional statistical indirect associations were calculated as a × b for men and (a+a_gender) × (b+b_gender) for women. Confidence intervals were obtained from 5,000 multiplier-bootstrap replications using the same multiplier for the mediator and outcome models; the random seed was 2,026. In the seventh wave, multiplier resampling was performed at the primary sampling unit (PSU) level; in the sixth wave, individual observations were treated as resampling units because public-use PSU identifiers were unavailable. Because the data are cross-sectional, these quantities are described as statistical indirect associations rather than causal mediation effects.

To evaluate whether small gender differences in the linear slopes were practically negligible rather than merely non-significant, we conducted *post hoc* two one-sided tests. The smallest effect size of interest was defined as a 0.10-standard-deviation difference between genders across the full four-unit exposure range from never to daily. Equivalence was concluded when the 90% confidence interval for the relevant interaction or indirect-association difference fell within the corresponding ± margin. Variance inflation factors were calculated after centering all continuous variables involved in interactions; the maximum variance inflation factor (VIF) was 2.27.

Secondary analyses treated ICT use as a five-category variable, jointly tested the four category-by-gender terms, applied Holm adjustment to category-specific interactions, estimated marginal standardized means, and repeated the analysis separately in each wave. The several-times-per-month interaction was further evaluated using category-by-gender-by-wave terms because the pooled categorical analysis indicated a localized deviation from the overall linear pattern.

Sensitivity analyses used the original five-item composite, family-to-work interference, and work-related rumination as alternate mediators. The five-item composite retained the common primary sample, whereas FIW and rumination models used mediator-specific complete cases. Additional analyses used the original five-item composite to examine daily vs. less frequent ICT use, expanded paid-work covariates, separate wave models, ICT-by-wave interactions, and a seventh-wave model additionally controlling marital status and children. Because the exposure originated in the work domain and the measurement analyses supported directional scoring, WIF was used as the primary mediator; the original five-item composite remained a sensitivity measure.

As a design-based sensitivity analysis, the seventh-wave models were re-estimated with the survey weight, 55 strata, and 4,636 nested primary sampling units using the R (survey package; R Foundation for Statistical Computing, Vienna, Austria). Descriptive statistics were weighted; unweighted counts and standardized mean differences were reported. Complete-case selection was examined by comparing included and excluded wage workers. Analyses were performed in Google Colab using Python 3.12 (Python Software Foundation, Wilmington, DE, USA) (pandas and statsmodels) and R (survey version 4.5 and lavaan). All conventional tests were two-sided with *p* < 0.05, while equivalence tests used two one-sided tests at α = 0.05.

The KWCS is publicly available and fully de-identified. Ethical review and additional informed consent were therefore not required for this secondary analysis in accordance with applicable institutional and national requirements.

## 3 Results

### 3.1 Sample derivation and descriptive characteristics

The final sample comprised 56,640 wage workers: 26,536 men (46.8%) and 30,104 women (53.2%). Weighted mean after-hours ICT use was 2.25 for men and 2.11 for women [standardized mean difference (SMD) = −0.10]. WIF was nearly identical before adjustment (2.08 in both groups; SMD < 0.01), whereas women had slightly higher exhaustion (2.76 vs. 2.73; SMD = 0.04) and markedly fewer paid working hours (36.24 vs. 41.15; SMD = −0.45). Education coded from the detailed seven-level variable showed bachelor's degree or above in 45.6% of men and 37.3% of women. Women were more frequently in service and sales occupations, whereas men were more frequently in manual or agricultural occupations. The largest category-level SMDs concerned occupation and employment status (Table 1). Among 63,213 wage workers before complete-case selection, pooled missingness was 2.79% for ICT use, 3.71% for WIF, 0.12% for exhaustion, 0.13% for education, and 3.73% for weekly hours; wave-specific percentages are reported in Supplementary Table S3. Included and excluded wage workers differed by SMDs of 0.03 for ICT use, 0.06 for the five-item composite, 0.08 for exhaustion, and 0.14 for weekly hours.

**Table 1:** Weighted characteristics of the analytic sample by gender.

Variable	Men	Women	SMD
Unweighted *N*	26,536	30,104	—
Continuous variables, weighted mean (SD)			
After-hours ICT use	2.25 (1.43)	2.11 (1.41)	−0.10
Work-to-family interference	2.08 (0.91)	2.08 (0.90)	< 0.01
Family-to-work interference	1.71 (0.78)	1.75 (0.80)	0.06
Five-item work–family composite	1.93 (0.75)	1.93 (0.76)	−0.01
Exhaustion	2.73 (0.89)	2.76 (0.88)	0.04
Weekly paid working hours	41.15 (10.20)	36.24 (11.74)	−0.45
Age group, unweighted *n* (weighted %)			
15–19	103 (0.5%)	120 (0.7%)	0.03
20–29	3,156 (13.5%)	3,421 (19.2%)	0.15
30–39	6,284 (24.4%)	5,830 (20.2%)	−0.10
40–49	6,438 (25.5%)	6,631 (22.6%)	−0.07
50–59	5,861 (21.8%)	7,934 (21.8%)	< 0.01
≥60	4,694 (14.3%)	6,168 (15.6%)	0.04
Education, unweighted *n* (weighted %)			
High school or below	10,184 (36.4%)	14,497 (41.6%)	0.11
Associate degree	4,833 (18.0%)	6,014 (21.1%)	0.08
Bachelor's degree or above	11,519 (45.6%)	9,593 (37.3%)	−0.17
Occupation, unweighted *n* (weighted %)			
White-collar (KSCO 1–3)	11,647 (45.7%)	13,566 (51.3%)	0.11
Service and sales (KSCO 4–5)	3,626 (10.3%)	9,985 (26.0%)	0.42
Manual/agricultural (KSCO 6–9)	11,180 (43.7%)	6,545 (22.7%)	−0.46
Military (KSCO 10)	83 (0.3%)	8 (< 0.1%)	−0.07
Employment status, unweighted *n* (weighted %)			
Permanent	22,011 (81.6%)	22,855 (73.5%)	−0.19
Temporary	2,953 (12.4%)	6,196 (22.8%)	0.27
Daily	1,572 (6.0%)	1,053 (3.6%)	−0.11

Counts are unweighted; means, standard deviations, and percentages are weighted using wave-specific standardized weights. After-hours ICT use ranged from 1 (never) to 5 (daily). SMD, standardized mean difference, calculated as women minus men; KSCO, Korean Standard Classification of Occupations.

### 3.2 Measurement properties

The WIF items had an inter-item correlation of 0.695 and a Spearman–Brown reliability of 0.820. Corresponding Spearman–Brown coefficients were 0.906 for family-to-work interference and 0.902 for exhaustion. The five-item composite had α = 0.882 and approximate ω = 0.882. A four-item two-factor WIF/family-to-work model fit substantially better [comparative fit index (CFI) > 0.999, Tucker-Lewis index (TLI) > 0.999, root mean square error of approximation (RMSEA) = 0.012, standardized root mean square residual (SRMR) = 0.001] than the five-item one-factor model (CFI = 0.982, RMSEA = 0.242) or four-item one-factor model (CFI = 0.987, RMSEA = 0.316). Because each directional factor contained only two indicators and the model had very few degrees of freedom, these fit indices were interpreted as supportive rather than definitive. Five-item-model invariance diagnostics showed small changes in comparative fit across gender and wave, but poor absolute fit limited strong claims of measurement invariance. WIF was therefore retained as the primary mediator, with the five-item composite and other components as sensitivity measures (Supplementary Tables S1, S2).

### 3.3 Primary weighted associations

In the weighted primary mediator model, each one-category increase in after-hours ICT use was associated with 0.064 higher WIF (95% CI 0.053–0.074; standardized β = 0.10). The ICT-by-gender interaction was small and not conventionally significant (B = −0.009, 95% CI −0.023 to 0.006; *p* = 0.237). In the outcome model, WIF was associated with exhaustion (B = 0.329, 95% CI 0.314–0.344; β = 0.34), and the WIF-by-gender interaction was not significant (B = −0.009, 95% CI −0.031 to 0.012; *p* = 0.379). After WIF was included, a small direct association remained between ICT use and exhaustion (B = 0.021, 95% CI 0.011–0.030; β = 0.03), with no ICT-by-gender interaction (B = 0.005, 95% CI −0.008 to 0.019; *p* = 0.450). The total ICT–exhaustion association was B = 0.042 (95% CI 0.031–0.052; β = 0.07), and its interaction with gender was B = 0.002 (95% CI −0.013 to 0.016; *p* = 0.808). Model adjusted *R*^2^ values were 0.068 for WIF, 0.157 for exhaustion with WIF, and 0.053 for the total-association model (Table 2; full coefficients in Supplementary Table S4).

**Table 2:** Primary weighted linear models for work-to-family interference and exhaustion.

Model and term	B	SE	95% CI	*P*-value	Standardized β
Mediator model: WIF					
After-hours ICT use (centered)	0.064	0.005	0.053–0.074	< 0.001	0.10
Gender: women vs. men	0.091	0.011	0.069–0.112	< 0.001	0.10
ICT use × gender	−0.009	0.007	−0.023 to 0.006	0.237	−0.01
Weekly hours (centered)	0.014	< 0.001	0.013–0.015	< 0.001	0.18
Adjusted *R*^2^	0.068				
Outcome model: exhaustion with WIF					
After-hours ICT use (centered)	0.021	0.005	0.011–0.030	< 0.001	0.03
Work-to-family interference (centered)	0.329	0.008	0.314–0.344	< 0.001	0.34
Gender: women vs. men	0.054	0.010	0.034–0.074	< 0.001	0.06
ICT use × gender	0.005	0.007	−0.008 to 0.019	0.450	0.01
WIF × gender	−0.009	0.011	−0.031 to 0.012	0.379	−0.01
Weekly hours (centered)	0.004	< 0.001	0.004–0.005	< 0.001	0.06
Adjusted *R*^2^	0.157				
Total-association model: exhaustion					
After-hours ICT use (centered)	0.042	0.005	0.031–0.052	< 0.001	0.07
Gender: women vs. men	0.083	0.011	0.062–0.105	< 0.001	0.09
ICT use × gender	0.002	0.007	−0.013 to 0.016	0.808	< 0.01
Adjusted *R*^2^	0.053				

N = 56,640. Models used wave-specific standardized weights normalized within wave and HC3 robust standard errors. Continuous variables involved in interactions were weighted-mean centered. Models adjusted for age, education, detailed KSCO occupation, weekly paid working hours, and wave. Standardized β values were calculated using weighted standard deviations. Full covariate coefficients appear in Supplementary Table S4.

### 3.4 Conditional indirect associations and *post hoc* equivalence analyses

The conditional statistical indirect association through WIF was 0.021 for men (95% bootstrap CI 0.017–0.025) and 0.018 for women (95% bootstrap CI 0.014–0.021). The women-minus-men difference was −0.003 (95% bootstrap CI −0.008 to 0.002), and the interval included zero. The 90% CI for the difference (−0.008 to 0.001) was contained within the ±0.022 margin. Under the stated *post hoc* criterion, the overall linear ICT-by-gender interactions in the WIF and total-exhaustion models and the gender difference in the indirect association met the corresponding 0.10-SD full-range criteria (Table 3).

**Table 3:** Conditional indirect associations and *post hoc* practical-equivalence analyses.

Effect	Estimate	95% CI	90% CI	Equivalence margin	Conclusion
Men: ICT → WIF → exhaustion	0.021	0.017–0.025	0.018–0.024	—	CI excludes zero
Women: ICT → WIF → exhaustion	0.018	0.014–0.021	0.015–0.021	—	CI excludes zero
Women – men indirect association	−0.003	−0.008 to 0.002	−0.008 to 0.001	±0.022	Includes zero; met *post hoc* criterion
ICT × gender in WIF model	−0.009	−0.023 to 0.006	−0.021 to 0.003	±0.023	Met *post hoc* criterion
ICT × gender in total exhaustion model	0.002	−0.013 to 0.016	−0.010 to 0.014	±0.022	Met *post hoc* criterion

N = 56,640. Conditional indirect associations and their women-minus-men difference were estimated using 5,000 multiplier-bootstrap replications (seed = 2,026). Confidence intervals for the regression interaction coefficients were based on HC3 robust standard errors. The women-minus-men 95% confidence interval includes zero. *Post hoc* equivalence margins correspond to a 0.10-SD gender difference across the full Never-to-Daily exposure range and were evaluated with 90% confidence intervals.

### 3.5 Covariate-standardized gender differences

**Figure 1:**
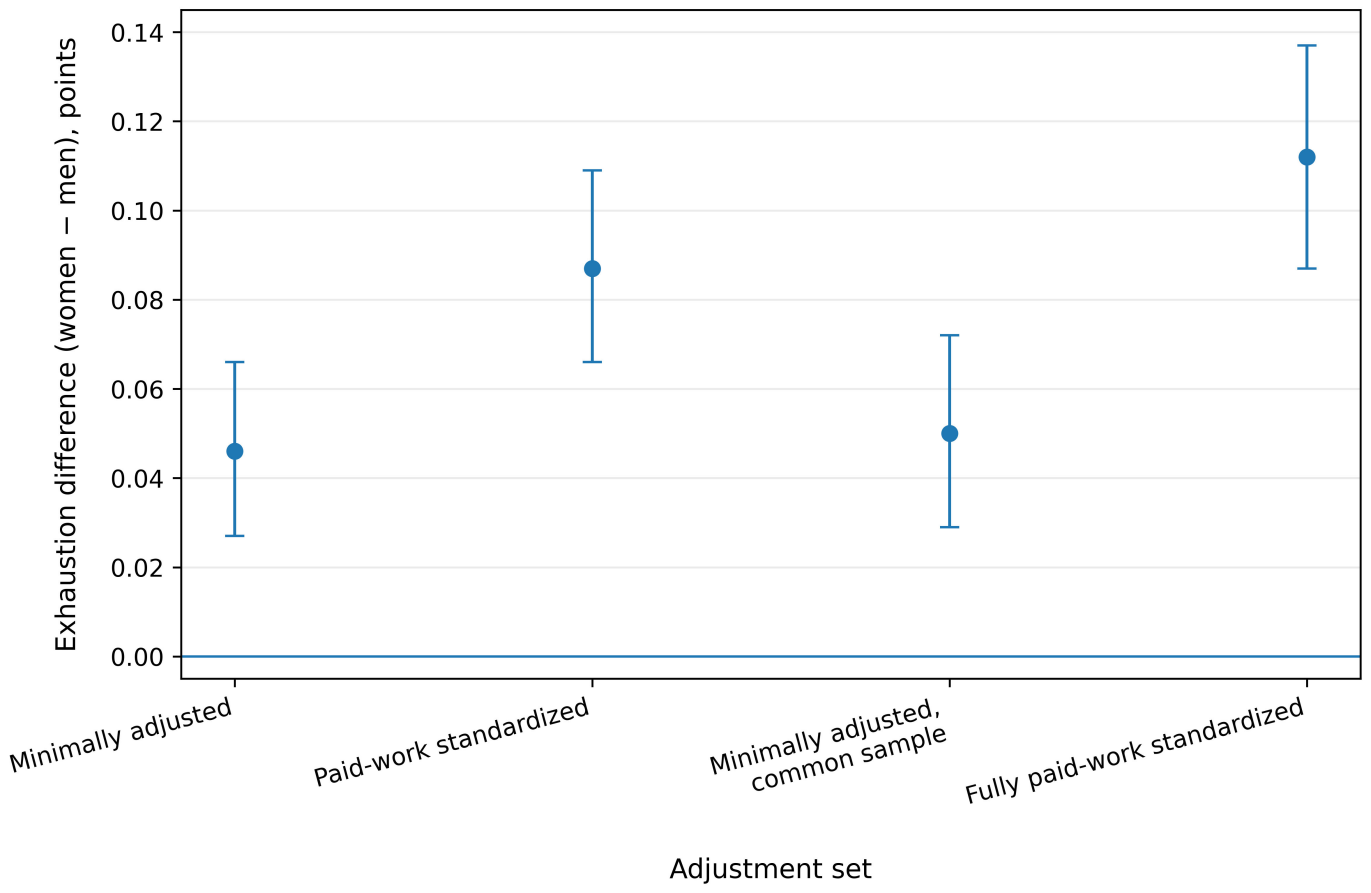
Adjusted women-minus-men exhaustion differences across adjustment sets. Minimal models adjusted for age, education, and wave. Paid-work-standardized models additionally standardized occupation and weekly paid hours. The fully standardized common-sample model additionally included employment status, industry, shift/night work, supervisory responsibility, household income adequacy, schedule control, part-time status, and workplace size.

The weighted unadjusted women-minus-men differences were −0.004 for WIF and 0.038 for exhaustion. After adjustment for age, education, and wave, the standardized differences were 0.021 for WIF (95% CI 0.002–0.041) and 0.046 for exhaustion (95% CI 0.027–0.066). Standardizing additionally to a common distribution of occupation and paid working hours increased the conditional differences to 0.096 for WIF (95% CI 0.075–0.117) and 0.087 for exhaustion (95% CI 0.066–0.109). In a common *N* = 46,431 sample additionally standardized for employment status, industry, shift and night work, supervisory responsibility, household income adequacy, schedule control, part-time status, and workplace size, the exhaustion difference was 0.112 (95% CI 0.087–0.137). A sequential regression diagnostic using the original five-item composite showed that the women coefficient changed from −0.004 in the gender-only model to 0.018 after adding ICT, demographics, wave, and occupation, and to 0.054 after adding paid working hours. For exhaustion, the corresponding coefficient changed from 0.038 to 0.047 and then to 0.083; it was 0.061 after the five-item composite was additionally included. These are covariate-standardized or conditional contrasts, not estimates of pre-exposure baseline differences or total causal effects of gender (Figure 1; Supplementary Table S5).

### 3.6 Categorical and wave-specific secondary analyses

**Figure 2:**
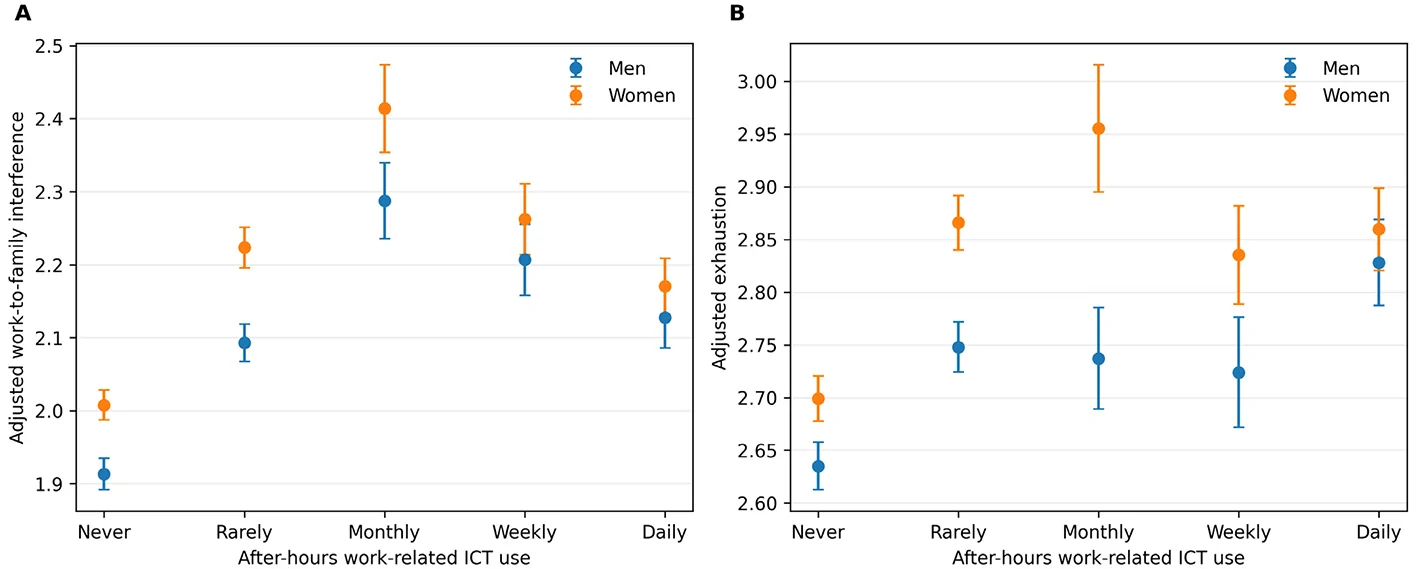
Adjusted work-to-family interference and exhaustion by after-hours ICT-use category and gender. Points are marginal standardized means with 95% confidence intervals from weighted categorical models. Categories were modeled without imposing equal spacing. **(A)** Work-to-family interference. **(B)** Exhaustion.

The five-category analysis indicated departure from a simple linear dose–response pattern (Figure 2). The joint ICT-category-by-gender test was not significant for WIF (4-df Wald *p* = 0.073), and none of the category-specific WIF interactions remained significant after Holm correction. For total exhaustion, however, the joint categorical interaction was significant (*p* < 0.001). The largest localized difference occurred among workers using ICT several times per month: the women-minus-men interaction relative to never users was B = 0.154 (Holm-adjusted *p* = 0.001), whereas the daily-use interaction was not significant. This exploratory monthly-use pattern was observed in both waves. The additional women-minus-men exhaustion contrast was B = 0.166 in the sixth wave (95% CI 0.033–0.299; *p* = 0.014) and B = 0.162 in the seventh wave (95% CI 0.064–0.260; *p* = 0.001). Neither the monthly category-specific three-way interaction with wave (*p* = 0.967) nor the four-degree-of-freedom category-by-gender-by-wave test (*p* = 0.928) indicated detectable wave heterogeneity. The monthly WIF interaction was not significant in either wave, and the localized exhaustion difference remained after adjustment for WIF in both waves (Supplementary Table S6 and Supplementary Figure S2).

### 3.7 Sensitivity analyses

Alternate-mediator analyses were consistent with the primary interpretation. ICT use was positively associated with the five-item composite (B = 0.056), FIW (B = 0.039), and rumination (B = 0.078); none of their linear ICT-by-gender or mediator-by-gender interactions was statistically significant. In a daily-vs.-less-frequent five-item-composite sensitivity analysis, daily use was associated with higher composite scores (B = 0.089, 95% CI 0.052–0.125) and a remaining direct association with exhaustion (B = 0.101, 95% CI 0.061–0.141); the corresponding gender interactions were not significant. Expanded-covariate and seventh-wave family-covariate analyses using the original five-item composite were consistent with the broader interpretation regarding association direction and the absence of statistically significant gender moderation. In wave-specific five-item-composite models, the ICT coefficient was 0.059 in the sixth wave and 0.048 in the seventh; the pooled ICT-by-wave interaction was small but significant for the composite (B = −0.015, *p* = 0.016), whereas it was not significant for the WFC5-adjusted direct association with exhaustion (*p* = 0.898). In the fully design-adjusted seventh-wave WIF analysis (*N* = 25,783; 55 strata; 4,636 nested primary sampling units), ICT use was associated with WIF (B = 0.058, 95% CI 0.039–0.077), WIF was associated with exhaustion (B = 0.290, 95% CI 0.268–0.312), and the total ICT–exhaustion association was B = 0.036 (95% CI 0.020–0.052). The ICT-by-gender interactions for WIF (*p* = 0.141), the direct association (*p* = 0.182), and the total association (*p* = 0.414), as well as the WIF-by-gender interaction (*p* = 0.516), were not significant (Supplementary Tables S6–S8).

## 4 Discussion

This study used two recent KWCS waves to examine after-hours ICT use, directional work-to-family interference, exhaustion, and gender. Three primary findings emerged. First, more frequent after-hours ICT use was associated with higher WIF and exhaustion, with a positive statistical indirect association through WIF. Second, the ICT-by-gender interactions in the WIF and total-exhaustion models and the gender difference in the indirect association met the stated *post hoc* equivalence criteria; the remaining path interactions were not statistically significant. Third, women showed higher covariate-standardized exhaustion levels, although this contrast depended on the adjustment set and should not be interpreted as a longitudinal pre-exposure baseline. Exploratory categorical analyses additionally identified a localized, non-monotonic gender difference in exhaustion at several-times-per-month use.

The primary findings are consistent with boundary theory and resource-based perspectives on work–family dynamics. After-hours work-related communication may increase the permeability of boundaries between work and non-work domains and facilitate frequent or unplanned role transitions (13–15). Sustained connectivity may also consume time, attention, and energy that would otherwise support recovery (16, 19) and may prolong cognitive and emotional engagement with work after formal working hours (20–23). The directional WIF measure aligned the work-originating exposure with interference entering family or personal life, and its strong association with exhaustion is consistent with meta-analytic evidence identifying work-to-family conflict as a robust correlate of emotional exhaustion and related strain outcomes (32, 39), as well as with the conceptualization of exhaustion as a central energetic component of burnout (38). The two-factor measurement results also indicate that combining work-to-family and family-to-work items into a single score obscures meaningful directionality. Because the data are cross-sectional, however, these theoretical processes remain interpretations rather than established temporal mechanisms.

After WIF was included, a small association between after-hours ICT use and exhaustion remained. This residual association does not identify a causal direct effect, but it is compatible with additional processes such as impaired psychological detachment, sustained work-related rumination, cognitive load, and organizational expectations of availability (20–23, 29, 40). Korean studies have also reported associations of sudden work recall with psychological health problems (9) and of work-related activities during free time with sleep disturbance (10). The localized monthly-use gender divergence was likewise not fully accounted for by the measured WIF pathway, underscoring that the single frequency item may combine heterogeneous work contexts. Longitudinal and intensive repeated-measures studies are needed to determine how these processes unfold over time.

The *post hoc* equivalence analyses provide additional context for the overall linear gender pattern rather than relying on *p* > 0.05 alone. The 90% confidence intervals for the ICT-by-gender interactions in the WIF and total-exhaustion models and for the indirect-association difference were contained within margins corresponding to 0.10 outcome standard deviations across the full exposure range. Thus, these quantities met the stated *post hoc* criteria; this does not establish equivalence for the other path interactions or at every exposure category. The exploratory categorical analysis showed a localized divergence in exhaustion at monthly use that was observed in both waves without detectable wave heterogeneity and was not fully accounted for by measured WIF. The non-monotonic pattern may reflect heterogeneous organizational contexts—for example, episodic but unpredictable demands, concentrated deadlines, or availability expectations—that cannot be identified with the single survey item. This result should therefore be interpreted as hypothesis-generating rather than as evidence for a female-specific dose–response.

The adjusted gender-level findings also require care. WIF was nearly identical before adjustment, and women reported fewer paid hours. The conditional difference increased when occupation and paid hours were standardized, illustrating that these variables are not neutral confounders: they are themselves features of gendered work arrangements and may act as mediators, suppressors, or proxies for unmeasured conditions. The sequential diagnostic showed that the adjusted coefficient increased most clearly after paid working hours were added. We therefore present minimally adjusted and paid-work-standardized contrasts as distinct estimands rather than treating one as the uniquely correct gender effect. The higher covariate-standardized exhaustion among women is compatible with—but does not demonstrate—contributions from unpaid care, emotional labor, job demands, income inequality, schedule control, or differential recovery opportunities (35, 36). Cross-national time-use research has documented that employed women often perform a larger share of childcare and household work (33, 34), and women in the present sample reported higher exhaustion despite fewer paid working hours. Nevertheless, the KWCS measures used here did not directly assess unpaid care, household labor, emotional labor, or organizational availability expectations, so direct measurement of these factors is needed before attributing the difference to a specific structural mechanism.

Although the coefficients were statistically precise, most technology-use effects were small in absolute magnitude. A one-category increase in ICT use corresponded to approximately 0.10 SD higher WIF and 0.07 SD higher exhaustion in the total-association model, whereas the remaining direct association was approximately 0.03 SD. The WIF–exhaustion association was larger (approximately 0.34 SD). These magnitudes caution against portraying after-hours connectivity as a single dominant cause of exhaustion. Nevertheless, small individual-level associations may remain relevant when exposure is widespread, and the categorical findings indicate that linear averages can conceal subgroup-specific patterns.

The policy implications should likewise be proportionate to the observational evidence. Clear expectations about non-work-time communication and right-to-disconnect approaches may support recovery across employees (12, 41, 42), and supervisor and organizational support may help reduce work–family conflict (43, 44), but this study does not establish that a particular legal or organizational intervention will reduce exhaustion. The findings support considering after-hours connectivity within occupational psychosocial risk assessment. Because women's covariate-standardized exhaustion remained higher, connectivity-focused measures may need to be considered alongside predictable scheduling, family-supportive supervision, affordable childcare, pay equity, and more equitable care arrangements. The broadly similar overall linear associations support general connectivity protections, while the adjusted level difference warrants continued gender-responsive monitoring.

This study has several strengths. It used a large sample from two recent waves of a nationwide working-conditions survey and applied standardized survey weights in the pooled analysis, with a fully design-adjusted seventh-wave sensitivity analysis. It evaluated directional work-to-family interference, tested gender moderation across all modeled paths, reported standardized coefficients and equivalence tests, and examined categorical and wave-specific patterns. It also separated two conceptually distinct questions: whether associations varied by gender and whether covariate-standardized outcome levels differed between men and women. Robust standard errors and bootstrap confidence intervals were used to reduce reliance on conventional distributional assumptions, and the original five-item composite and expanded covariate models were retained as sensitivity analyses.

Several limitations remain. First, the cross-sectional design does not establish temporal order, and cross-sectional indirect-association estimates can be biased representations of longitudinal mediation processes (45); reverse and reciprocal associations are possible. Second, the exposure, mediator, and outcome were self-reported, so reporting bias and common-method variance may have inflated their associations. Third, the single ICT frequency item did not measure duration, voluntariness, content, urgency, or organizational response norms. Fourth, WIF and exhaustion were each based on two items. One WIF item explicitly referred to being too tired after work to complete household tasks, creating conceptual overlap with exhaustion that may have strengthened the WIF–exhaustion association. Although reliability was good and the directional two-factor specification fit better than one-factor alternatives, each factor had only two indicators and very few degrees of freedom; these measures are not comprehensive clinical or burnout instruments. Fifth, the binary gender variable did not represent gender diversity. Sixth, unpaid care, emotional labor, detailed job demands, dependent-child age, household composition, and actual communication logs were not harmonized across waves. Seventh, complete-case analysis may have introduced selection bias, although included-vs.-excluded SMDs were generally small. Eighth, the sixth public-use file lacked full stratum and primary sampling unit identifiers; the pooled primary analysis therefore combined standardized weights with HC3 standard errors, while complete design correction was possible only for the seventh-wave sensitivity analysis. Ninth, the two waves were independent cross-sections, which precluded assessment of within-person change. Finally, the localized monthly finding emerged from secondary categorical analyses and should be replicated prospectively, and the findings may not generalize to self-employed workers or to other institutional and cultural contexts.

## 5 Conclusion

After-hours work-related ICT use was associated with greater WIF and exhaustion among Korean wage workers, with a positive statistical indirect association through WIF. The ICT-by-gender interactions in the WIF and total-association models and the gender difference in the indirect association met the stated *post hoc* equivalence criteria, while women had higher covariate-standardized exhaustion levels. Exploratory categorical analyses additionally suggested a localized gender difference in exhaustion among workers using ICT several times per month; the pattern was observed in both waves without detectable wave heterogeneity but was not explained by the measured WIF pathway. These results support consideration of after-hours connectivity in occupational psychosocial risk management, while connectivity-focused measures should not be assumed to address all determinants of gender differences in exhaustion. Future KWCS waves would benefit from time-use diaries, care-burden modules, detailed availability expectations, and repeated measurements capable of establishing temporal order.

## Data Availability

Publicly available datasets were analyzed in this study. The Korean Working Conditions Survey data can be obtained from the Occupational Safety and Health Research Institute (OSHRI), Korea Occupational Safety and Health Agency (https://oshri.kosha.or.kr). Analytic code is available from the corresponding author upon reasonable request.
