# Supplementary material for: After-hours work-related technology use, work-to-family interference, and exhaustion among Korean wage workers: gender patterns in associations and covariate-standardized levels
Source: Front Public Health. 2026 Aug 21;14:1939418. doi: 10.3389/fpubh.2026.1939418 (PMC13541763; doi:10.3389/fpubh.2026.1939418)
Supplement: Supplementary file 1 [file Supplementary_file_1.docx]

**Supplementary Material**

**Supplementary Figure S1. Participant flow.**

*Note. Exclusions are sequential. The final primary sample required valid responses to all five work–family items so that the WIF primary analysis and five-item-composite sensitivity were directly comparable.*

**Supplementary Table S1. Item wording, scoring, and scale assignment.**

| **Construct/item** | **Verbatim item** | **Response options** | **Scoring/use** |
| --- | --- | --- | --- |
| After-hours ICT use | In the last month, in your free time, how often have you used communication tools for work? | Daily; several times/week; several times/month; rarely; never | Reverse-coded 1–5; higher = more frequent |
| Work rumination | Kept worrying about work when you were not working | Always to never | Reverse-coded; sensitivity measure |
| WIF item 1 | Felt too tired after work to do household jobs that needed to be done | Always to never | Reverse-coded |
| WIF item 2 | Job prevented you from giving the time you wanted to your family | Always to never | Reverse-coded |
| FIW item 1 | Difficult to concentrate on your job because of family responsibilities | Always to never | Reverse-coded |
| FIW item 2 | Family responsibilities prevented you from giving the time you should to your job | Always to never | Reverse-coded |
| Exhaustion item 1 | I feel physically exhausted at the end of the working day | Always to never | Reverse-coded |
| Exhaustion item 2 | I feel emotionally drained by my work | Always to never | Reverse-coded |

*Note. The same item wording and response structure were used in both survey waves. WIF = work-to-family interference; FIW = family-to-work interference.*

**Supplementary Table S2. Reliability, dimensionality, and invariance diagnostics.**

| **Scale/model** | **Result** | **Interpretation** |
| --- | --- | --- |
| WIF (2 items) | Inter-item r = 0.695; Spearman–Brown = 0.820 | Primary mediator; acceptable two-item reliability |
| FIW (2 items) | Inter-item r = 0.829; Spearman–Brown = 0.906 | Sensitivity mediator |
| Exhaustion (2 items) | Inter-item r = 0.821; Spearman–Brown = 0.902 | Outcome; not a full burnout inventory |
| Five-item composite | α = 0.882; approximate ω = 0.882 | Sensitivity mediator |
| Five-item one-factor CFA | CFI = 0.982; TLI = 0.964; RMSEA = 0.242; SRMR = 0.059 | Poor absolute fit |
| Four-item one-factor CFA | CFI = 0.987; TLI = 0.962; RMSEA = 0.316; SRMR = 0.064 | Poor absolute fit |
| Four-item WIF/FIW two-factor CFA | CFI > 0.999; TLI > 0.999; RMSEA = 0.012; SRMR = 0.001 | Substantially better fit; interpreted cautiously because each factor had two indicators and the model had very few degrees of freedom |
| Gender invariance diagnostics, five-item model | ΔCFI = 0.003 (metric), <0.001 (scalar) | Small comparative-fit changes, but poor absolute fit limits strong invariance claims |
| Wave invariance diagnostics, five-item model | ΔCFI < 0.001 (metric), 0.002 (scalar) | Small comparative-fit changes, but poor absolute fit limits strong invariance claims |

*Note. Ordinal WLSMV models were used for CFA. The two-factor fit supports directional scoring but does not by itself establish definitive construct validity or invariance for a two-indicator WIF factor.*

**Supplementary Table S3. Sample derivation, variable-level missingness, and included-versus-excluded comparison.**

**Panel A. Sequential sample derivation**

| **Selection stage** | **Remaining N** | **Excluded at step** |
| --- | --- | --- |
| Raw pooled sample | 100,733 | 0 |
| Wage workers (employment-type code 3) | 63,213 | 37,520 |
| + valid after-hours ICT | 61,450 | 1,763 |
| + complete five work–family items | 58,650 | 2,800 |
| + valid exhaustion | 58,603 | 47 |
| Final primary complete-case sample | 56,640 | 1,963 |

**Panel B. Missingness among wage workers, n (%)**

| **Variable** | **6th wave (N=33,063)** | **7th wave (N=30,150)** | **Pooled (N=63,213)** |
| --- | --- | --- | --- |
| After-hours ICT | 55 (0.17%) | 1,708 (5.67%) | 1,763 (2.79%) |
| Five-item composite | 1,969 (5.96%) | 1,118 (3.71%) | 3,087 (4.88%) |
| WIF | 1,476 (4.46%) | 872 (2.89%) | 2,348 (3.71%) |
| FIW | 1,052 (3.18%) | 699 (2.32%) | 1,751 (2.77%) |
| Exhaustion | 46 (0.14%) | 31 (0.10%) | 77 (0.12%) |
| Gender | 0 (0.00%) | 0 (0.00%) | 0 (0.00%) |
| Age group | 0 (0.00%) | 0 (0.00%) | 0 (0.00%) |
| Education | 53 (0.16%) | 27 (0.09%) | 80 (0.13%) |
| Occupation | 0 (0.00%) | 0 (0.00%) | 0 (0.00%) |
| Weekly paid hours | 163 (0.49%) | 2,196 (7.28%) | 2,359 (3.73%) |
| Employment status | 0 (0.00%) | 0 (0.00%) | 0 (0.00%) |
| Industry | 0 (0.00%) | 0 (0.00%) | 0 (0.00%) |
| Shift work | 112 (0.34%) | 51 (0.17%) | 163 (0.26%) |
| Night work | 79 (0.24%) | 98 (0.33%) | 177 (0.28%) |
| Supervisory responsibility | 1,555 (4.70%) | 2,235 (7.41%) | 3,790 (6.00%) |
| Household income adequacy | 152 (0.46%) | 94 (0.31%) | 246 (0.39%) |
| Schedule control | 211 (0.64%) | 406 (1.35%) | 617 (0.98%) |
| Part-time status | 156 (0.47%) | 137 (0.45%) | 293 (0.46%) |
| Marital status | Not available | 541 (1.79%) | 7th-wave sensitivity only |
| Presence of children | Not available | 139 (0.46%) | 7th-wave sensitivity only |

**Panel C. Included-versus-excluded wage-worker comparison**

| **Variable** | **Included mean** | **Excluded mean** | **SMD** | **Included nonmissing** | **Excluded nonmissing** |
| --- | --- | --- | --- | --- | --- |
| ICT | 2.201 | 2.151 | 0.034 | 56,640 | 4,810 |
| Five-item work–family composite | 1.926 | 1.882 | 0.056 | 56,640 | 3,486 |
| Exhaustion | 2.751 | 2.674 | 0.083 | 56,640 | 6,496 |
| Weekly paid working hours | 38.662 | 36.831 | 0.144 | 56,640 | 4,214 |

*Note. Missingness denominators are wage workers before complete-case selection. Marital status and children were available only in the seventh wave. Included-versus-excluded SMDs compare the final primary sample with other wage workers having a nonmissing value for the variable shown.*

**Panel D. Harmonization and coding rules**

| **Variable** | **Source/harmonization** | **Final coding** |
| --- | --- | --- |
| Education | Detailed seven-level education variable used in both waves | 1–4 high school or below; 5 associate degree; 6–7 bachelor’s degree or above |
| Occupation | Original KSCO major group retained in regression | Display: 1–3 white-collar; 4–5 service/sales; 6–9 manual/agricultural; 10 military |
| Weight | 6th-wave standardized weight and 7th-wave standardized weight (WT3) | Normalized to mean 1 within wave before pooling |
| Wage worker | Employment-type code 3 in both waves | Self-employed, employers, unpaid family workers excluded |
| After-hours ICT | Same 5-category item in both waves | Reverse-coded 1 never to 5 daily; centered for linear models |

*Note. The detailed education variable, original KSCO major group, correct standardized weight, and wage-worker code were verified against both public-use codebooks before final analysis.*

**Supplementary Table S4. Full primary weighted regression models.**

**Panel A. Outcome: WIF (N = 56,640; adjusted R² = 0.068)**

| **Term** | **B** | **SE** | **95% CI** | **p** |
| --- | --- | --- | --- | --- |
| Intercept | 1.899 | 0.021 | 1.857 to 1.940 | <.001 |
| Age 15–19 | -0.243 | 0.057 | -0.356 to -0.130 | <.001 |
| Age 20–29 | -0.134 | 0.018 | -0.170 to -0.098 | <.001 |
| Age 40–49 | 0.020 | 0.015 | -0.010 to 0.050 | .184 |
| Age 50–59 | 0.012 | 0.016 | -0.020 to 0.044 | .454 |
| Age ≥60 | -0.061 | 0.020 | -0.100 to -0.022 | .002 |
| Associate degree | 0.007 | 0.016 | -0.024 to 0.038 | .661 |
| Bachelor’s degree or above | 0.014 | 0.016 | -0.017 to 0.046 | .371 |
| 7th wave | 0.251 | 0.010 | 0.231 to 0.271 | <.001 |
| Occupation: Managers | 0.067 | 0.061 | -0.053 to 0.187 | .271 |
| Occupation: Professionals | 0.061 | 0.015 | 0.032 to 0.091 | <.001 |
| Occupation: Service | 0.058 | 0.019 | 0.022 to 0.095 | .002 |
| Occupation: Sales | 0.026 | 0.019 | -0.010 to 0.062 | .161 |
| Occupation: Skilled agriculture | 0.082 | 0.103 | -0.120 to 0.284 | .425 |
| Occupation: Craft/trades | 0.107 | 0.023 | 0.062 to 0.153 | <.001 |
| Occupation: Operators/assemblers | 0.069 | 0.022 | 0.025 to 0.112 | .002 |
| Occupation: Elementary occupations | 0.032 | 0.020 | -0.008 to 0.072 | .120 |
| Occupation: Military | -0.201 | 0.085 | -0.368 to -0.034 | .019 |
| After-hours ICT use (centered) | 0.064 | 0.005 | 0.053 to 0.074 | <.001 |
| Gender: women | 0.091 | 0.011 | 0.069 to 0.112 | <.001 |
| ICT × gender | -0.009 | 0.007 | -0.023 to 0.006 | .237 |
| Weekly hours (centered) | 0.014 | 0.000 | 0.013 to 0.015 | <.001 |

**Panel B. Outcome: exhaustion with WIF (N = 56,640; adjusted R² = 0.157)**

| **Term** | **B** | **SE** | **95% CI** | **p** |
| --- | --- | --- | --- | --- |
| Intercept | 2.586 | 0.020 | 2.548 to 2.625 | <.001 |
| Age 15–19 | -0.105 | 0.089 | -0.280 to 0.069 | .236 |
| Age 20–29 | 0.009 | 0.018 | -0.026 to 0.045 | .600 |
| Age 40–49 | -0.032 | 0.014 | -0.059 to -0.005 | .020 |
| Age 50–59 | 0.009 | 0.014 | -0.019 to 0.037 | .537 |
| Age ≥60 | -0.084 | 0.018 | -0.119 to -0.048 | <.001 |
| Associate degree | -0.025 | 0.014 | -0.053 to 0.003 | .081 |
| Bachelor’s degree or above | -0.021 | 0.015 | -0.049 to 0.008 | .163 |
| 7th wave | 0.233 | 0.010 | 0.214 to 0.252 | <.001 |
| Occupation: Managers | -0.091 | 0.068 | -0.225 to 0.043 | .184 |
| Occupation: Professionals | 0.064 | 0.014 | 0.036 to 0.092 | <.001 |
| Occupation: Service | 0.137 | 0.018 | 0.101 to 0.172 | <.001 |
| Occupation: Sales | 0.033 | 0.018 | -0.002 to 0.068 | .066 |
| Occupation: Skilled agriculture | 0.147 | 0.068 | 0.015 to 0.279 | .029 |
| Occupation: Craft/trades | 0.121 | 0.020 | 0.081 to 0.161 | <.001 |
| Occupation: Operators/assemblers | 0.034 | 0.020 | -0.004 to 0.073 | .081 |
| Occupation: Elementary occupations | 0.091 | 0.019 | 0.053 to 0.128 | <.001 |
| Occupation: Military | -0.309 | 0.092 | -0.488 to -0.129 | <.001 |
| After-hours ICT use (centered) | 0.021 | 0.005 | 0.011 to 0.030 | <.001 |
| Gender: women | 0.054 | 0.010 | 0.034 to 0.074 | <.001 |
| ICT × gender | 0.005 | 0.007 | -0.008 to 0.019 | .450 |
| WIF (centered) | 0.329 | 0.008 | 0.314 to 0.344 | <.001 |
| WIF × gender | -0.009 | 0.011 | -0.031 to 0.012 | .379 |
| Weekly hours (centered) | 0.004 | 0.000 | 0.004 to 0.005 | <.001 |

**Panel C. Outcome: exhaustion, total-association model (N = 56,640; adjusted R² = 0.053)**

| **Term** | **B** | **SE** | **95% CI** | **p** |
| --- | --- | --- | --- | --- |
| Intercept | 2.527 | 0.021 | 2.485 to 2.569 | <.001 |
| Age 15–19 | -0.184 | 0.093 | -0.367 to -0.001 | .049 |
| Age 20–29 | -0.034 | 0.019 | -0.071 to 0.003 | .072 |
| Age 40–49 | -0.026 | 0.015 | -0.055 to 0.004 | .086 |
| Age 50–59 | 0.013 | 0.015 | -0.017 to 0.043 | .404 |
| Age ≥60 | -0.104 | 0.019 | -0.141 to -0.066 | <.001 |
| Associate degree | -0.023 | 0.015 | -0.053 to 0.007 | .133 |
| Bachelor’s degree or above | -0.016 | 0.016 | -0.047 to 0.015 | .318 |
| 7th wave | 0.315 | 0.010 | 0.295 to 0.334 | <.001 |
| Occupation: Managers | -0.069 | 0.064 | -0.195 to 0.058 | .286 |
| Occupation: Professionals | 0.084 | 0.015 | 0.054 to 0.114 | <.001 |
| Occupation: Service | 0.156 | 0.019 | 0.118 to 0.193 | <.001 |
| Occupation: Sales | 0.041 | 0.019 | 0.004 to 0.078 | .028 |
| Occupation: Skilled agriculture | 0.174 | 0.083 | 0.013 to 0.336 | .035 |
| Occupation: Craft/trades | 0.156 | 0.022 | 0.114 to 0.199 | <.001 |
| Occupation: Operators/assemblers | 0.057 | 0.022 | 0.014 to 0.100 | .010 |
| Occupation: Elementary occupations | 0.101 | 0.020 | 0.061 to 0.141 | <.001 |
| Occupation: Military | -0.374 | 0.099 | -0.569 to -0.180 | <.001 |
| After-hours ICT use (centered) | 0.042 | 0.005 | 0.031 to 0.052 | <.001 |
| Gender: women | 0.083 | 0.011 | 0.062 to 0.105 | <.001 |
| ICT × gender | 0.002 | 0.007 | -0.013 to 0.016 | .808 |
| Weekly hours (centered) | 0.009 | 0.001 | 0.008 to 0.010 | <.001 |

*Note. All models used wave-specific standardized weights normalized within wave and HC3 robust standard errors. Reference categories were age 30–39, high school or below, clerical occupation, men, and the sixth wave.*

**Supplementary Table S5. Sequential adjustment and covariate-standardized gender contrasts.**

**Panel A. Women-minus-men standardized outcome contrasts**

| **Outcome** | **Adjustment set** | **Difference** | **95% CI** | **N** |
| --- | --- | --- | --- | --- |
| WIF | Observed weighted | -0.004 | Not estimated | 56,640 |
| Exhaustion | Observed weighted | 0.038 | Not estimated | 56,640 |
| WIF | Minimally adjusted | 0.021 | 0.002 to 0.041 | 56,640 |
| WIF | Paid-work standardized | 0.096 | 0.075 to 0.117 | 56,640 |
| WIF | Minimally adjusted, common sample | 0.023 | 0.001 to 0.045 | 46,431 |
| WIF | Fully paid-work standardized | 0.129 | 0.104 to 0.153 | 46,431 |
| Exhaustion | Minimally adjusted | 0.046 | 0.027 to 0.066 | 56,640 |
| Exhaustion | Paid-work standardized | 0.087 | 0.066 to 0.109 | 56,640 |
| Exhaustion | Minimally adjusted, common sample | 0.050 | 0.028 to 0.072 | 46,431 |
| Exhaustion | Fully paid-work standardized | 0.112 | 0.087 to 0.137 | 46,431 |

**Panel B. Sequential gender coefficients in the original five-item-composite and exhaustion models**

| **Outcome** | **Sequential specification** | **Women coefficient B** | **95% CI** | **p** | **R²** | **N** |
| --- | --- | --- | --- | --- | --- | --- |
| Five-item composite | Gender only | −0.004 | −0.021 to 0.013 | .640 | 0.000 | 56,640 |
| Five-item composite | + ICT | 0.004 | −0.013 to 0.021 | .645 | 0.012 | 56,640 |
| Five-item composite | + age, education, wave | 0.014 | −0.003 to 0.030 | .103 | 0.054 | 56,640 |
| Five-item composite | + occupation | 0.018 | 0.000 to 0.036 | .045 | 0.056 | 56,640 |
| Five-item composite | + weekly paid hours | 0.054 | 0.036 to 0.072 | <.001 | 0.071 | 56,640 |
| Exhaustion | Gender only | 0.038 | 0.018 to 0.058 | <.001 | 0.000 | 56,640 |
| Exhaustion | + ICT | 0.044 | 0.024 to 0.064 | <.001 | 0.005 | 56,640 |
| Exhaustion | + age, education, wave | 0.042 | 0.022 to 0.061 | <.001 | 0.039 | 56,640 |
| Exhaustion | + occupation | 0.047 | 0.026 to 0.068 | <.001 | 0.042 | 56,640 |
| Exhaustion | + weekly paid hours | 0.083 | 0.062 to 0.105 | <.001 | 0.054 | 56,640 |
| Exhaustion | + five-item composite | 0.061 | 0.041 to 0.082 | <.001 | 0.165 | 56,640 |

*Note. Panel A reports marginal standardized contrasts and treats minimally adjusted and paid-work-standardized estimands separately. Panel B directly traces how the gender coefficient in the original five-item-composite analysis changed as covariates were added; occupation and paid working hours may be mediators, suppressors, or proxies rather than neutral confounders.*

**Supplementary Table S6. Categorical and wave-specific analyses.**

**Panel A. Categorical ICT-by-gender tests**

| **Outcome** | **Test/term** | **B** | **SE** | **p** | **Interpretation** |
| --- | --- | --- | --- | --- | --- |
| WIF | Four-category omnibus | — | — | .073 | No overall categorical moderation |
| Exhaustion | Four-category omnibus | — | — | <.001 | Overall categorical pattern differed |
| Exhaustion | Rarely × women | 0.054 | 0.023 | .060 | Holm-adjusted p |
| Exhaustion | Monthly × women | 0.154 | 0.042 | .001 | Holm-adjusted p |
| Exhaustion | Weekly × women | 0.048 | 0.039 | .440 | Holm-adjusted p |
| Exhaustion | Daily × women | −0.032 | 0.033 | .440 | Holm-adjusted p |

*Note. Category-specific p-values are Holm adjusted. Never use was the reference category. The omnibus test jointly assessed the four category-by-gender interaction terms.*

**Panel B. Linear five-item-composite sensitivity by wave**

| **Path/test** | **Wave** | **B** | **95% CI** | **p** |
| --- | --- | --- | --- | --- |
| ICT → five-item composite | 6th | 0.059 | 0.048 to 0.071 | <.001 |
| ICT × gender → five-item composite | 6th | −0.004 | −0.020 to 0.012 | .619 |
| ICT direct association with exhaustion | 6th | 0.018 | 0.004 to 0.031 | .012 |
| ICT × gender direct association | 6th | −0.001 | −0.020 to 0.018 | .947 |
| ICT → five-item composite | 7th | 0.048 | 0.035 to 0.061 | <.001 |
| ICT × gender → five-item composite | 7th | −0.001 | −0.019 to 0.017 | .927 |
| ICT direct association with exhaustion | 7th | 0.018 | 0.005 to 0.031 | .006 |
| ICT × gender direct association | 7th | 0.009 | −0.009 to 0.027 | .347 |
| ICT × wave → five-item composite | Pooled | −0.015 | −0.027 to −0.003 | .016 |
| ICT × wave → WFC5-adjusted exhaustion | Pooled | 0.001 | −0.012 to 0.014 | .898 |

**Panel C. Wave-specific estimates for the monthly-use gender interaction**

| **Outcome** | **Wave/test** | **B** | **95% CI** | **p** | **Interpretation** |
| --- | --- | --- | --- | --- | --- |
| WIF | 6th | 0.103 | −0.027 to 0.233 | .122 | Not significant |
| WIF | 7th | −0.019 | −0.127 to 0.090 | .734 | Not significant |
| Total exhaustion | 6th | 0.166 | 0.033 to 0.299 | .014 | Significant |
| Total exhaustion | 7th | 0.162 | 0.064 to 0.260 | .001 | Significant |
| WIF-adjusted exhaustion | 6th | 0.140 | 0.010 to 0.271 | .035 | Significant |
| WIF-adjusted exhaustion | 7th | 0.167 | 0.077 to 0.256 | <.001 | Significant |
| Total exhaustion | Monthly × gender × wave | −0.004 | −0.169 to 0.162 | .967 | No wave heterogeneity |
| Total exhaustion | Four-category gender × wave omnibus | — | — | .928 | No wave heterogeneity |

*Note. Panel B used the original five-item composite as a linear sensitivity analysis. The mediator coefficient was positive in both waves but modestly smaller in the seventh wave. Panel C presents wave-specific estimates for the exploratory monthly-use exhaustion interaction; the estimates were similar, and no detectable wave heterogeneity was observed.*

**Supplementary Figure S2. Wave-specific adjusted categorical predictions.**

*Note. Panels A–B show WIF; Panels C–D show total exhaustion. Points are weighted marginal standardized means with 95% confidence intervals. The monthly-use exhaustion divergence was similar across waves, whereas the monthly WIF interaction was not significant in either wave.*

**Supplementary Table S7. Alternate-mediator and additional sensitivity analyses.**

**Panel A. Alternate work–family mediators using mediator-specific complete cases**

| **Mediator** | **ICT → mediator B (95% CI; p)** | **ICT × gender B (95% CI; p)** | **Mediator × gender B (95% CI; p)** | **N** |
| --- | --- | --- | --- | --- |
| Five-item composite | 0.056 (0.048 to 0.065; <.001) | −0.003 (−0.014 to 0.009; .660) | −0.023 (−0.048 to 0.001; .059) | 56,640 |
| FIW | 0.039 (0.031 to 0.048; <.001) | 0.007 (−0.005 to 0.019; .263) | −0.011 (−0.033 to 0.012; .346) | 57,868 |
| Rumination | 0.078 (0.066 to 0.090; <.001) | −0.013 (−0.029 to 0.002; .091) | −0.018 (−0.039 to 0.003; .089) | 58,237 |

*Note. WIF was the primary mediator and is therefore not repeated in this alternate-mediator table. FIW and rumination analyses used mediator-specific complete cases; the five-item composite used the common primary sample.*

**Panel B. Expanded-covariate, family-covariate, and daily-use analyses using the five-item composite**

| **Analysis** | **Path/contrast** | **B or difference** | **95% CI** | **p** | **N** |
| --- | --- | --- | --- | --- | --- |
| Expanded paid-work covariates | ICT → composite | 0.039 | 0.030 to 0.048 | <.001 | 46,431 |
| Expanded paid-work covariates | ICT × gender → composite | 0.004 | −0.008 to 0.017 | .487 | 46,431 |
| Expanded paid-work covariates | Composite → exhaustion | 0.395 | 0.376 to 0.414 | <.001 | 46,431 |
| Expanded paid-work covariates | Composite × gender → exhaustion | −0.023 | −0.050 to 0.003 | .087 | 46,431 |
| Expanded paid-work covariates | ICT direct association | 0.016 | 0.005 to 0.026 | .004 | 46,431 |
| Expanded paid-work covariates | ICT × gender direct association | 0.005 | −0.010 to 0.020 | .505 | 46,431 |
| Expanded paid-work standardization | Adjusted exhaustion difference, women − men | 0.112 | 0.087 to 0.137 | <.001 | 46,431 |
| 7th-wave + marital status/children | ICT → composite | 0.047 | 0.034 to 0.060 | <.001 | 25,347 |
| 7th-wave + marital status/children | ICT × gender → composite | −0.000 | −0.018 to 0.018 | .976 | 25,347 |
| 7th-wave + marital status/children | Composite → exhaustion | 0.372 | 0.352 to 0.393 | <.001 | 25,347 |
| 7th-wave + marital status/children | Composite × gender → exhaustion | −0.013 | −0.042 to 0.016 | .372 | 25,347 |
| 7th-wave + marital status/children | ICT direct association | 0.018 | 0.005 to 0.031 | .005 | 25,347 |
| 7th-wave + marital status/children | ICT × gender direct association | 0.008 | −0.010 to 0.026 | .386 | 25,347 |
| Daily vs. less frequent | Daily use → composite | 0.089 | 0.052 to 0.125 | <.001 | 56,640 |
| Daily vs. less frequent | Daily use × gender → composite | −0.045 | −0.094 to 0.005 | .076 | 56,640 |
| Daily vs. less frequent | Daily-use direct association with exhaustion | 0.101 | 0.061 to 0.141 | <.001 | 56,640 |
| Daily vs. less frequent | Daily use × gender direct association | −0.035 | −0.091 to 0.020 | .211 | 56,640 |
| Daily vs. less frequent | Composite → exhaustion | 0.417 | 0.399 to 0.435 | <.001 | 56,640 |
| Daily vs. less frequent | Composite × gender → exhaustion | −0.022 | −0.046 to 0.002 | .078 | 56,640 |

*Note. These sensitivity analyses used the original five-item composite. Expanded covariates were employment status, industry, shift/night work, supervisory responsibility, household income adequacy, schedule control, part-time status, and workplace size. The 7th-wave family model additionally included marital status and children.*

**Supplementary Table S8. Seventh-wave fully design-adjusted WIF sensitivity analysis.**

| **Term** | **B** | **95% CI** | **p** |
| --- | --- | --- | --- |
| ICT → WIF | 0.058 | 0.039 to 0.077 | <.001 |
| ICT × gender → WIF | −0.015 | −0.036 to 0.005 | .141 |
| WIF → exhaustion | 0.290 | 0.268 to 0.312 | <.001 |
| WIF × gender → exhaustion | 0.009 | −0.017 to 0.034 | .516 |
| ICT direct association | 0.019 | 0.004 to 0.034 | .013 |
| ICT × gender direct association | 0.012 | −0.006 to 0.029 | .182 |
| ICT total association | 0.036 | 0.020 to 0.052 | <.001 |
| ICT × gender total association | 0.008 | −0.011 to 0.027 | .414 |

*Note. Design: N = 25,783; 55 strata; 4,636 nested primary sampling units; no singleton strata; design degrees of freedom = 4,581. Models used centered ICT and WIF and adjusted for age, education, detailed occupation, and weekly paid hours.*

**Supplementary Table S9. Weighted correlations among the core variables.**

| **Variable** | **ICT** | **WFC5** | **WIF** | **FIW** | **Exhaustion** |
| --- | --- | --- | --- | --- | --- |
| ICT | 1.000 | 0.110 | 0.100 | 0.072 | 0.064 |
| WFC5 | 0.110 | 1.000 | 0.910 | 0.876 | 0.377 |
| WIF | 0.100 | 0.910 | 1.000 | 0.663 | 0.365 |
| FIW | 0.072 | 0.876 | 0.663 | 1.000 | 0.331 |
| Exhaustion | 0.064 | 0.377 | 0.365 | 0.331 | 1.000 |

*Note. Pearson correlations used wave-normalized standardized weights. WFC5 = five-item work–family composite.*
