# Supplementary material for: After-hours work-related technology use, work-to-family interference, and exhaustion among Korean wage workers: gender patterns in associations and covariate-standardized levels
Source: Front Public Health. 2026 Aug 21;14:1939418. doi: 10.3389/fpubh.2026.1939418 (PMC13541763; doi:10.3389/fpubh.2026.1939418)
Supplement: Supplementary file 2 [file Table_1.docx]

**STROBE Checklist for Cross-Sectional Studies**

Manuscript: After-Hours Work-Related Technology Use, Work-to-Family Interference, and Exhaustion Among Korean Wage Workers: Gender Patterns in Associations and Covariate-Standardized Levels

| **Item** | **No.** | **STROBE recommendation** | **Location in revised manuscript** |
| --- | --- | --- | --- |
| Title and abstract | 1 | Indicate the study’s design with a commonly used term in the title or abstract; provide an informative and balanced summary of what was done and found. | Title; Abstract |
| Background/rationale | 2 | Explain the scientific background and rationale for the investigation being reported. | Introduction, paragraphs 1–5 |
| Objectives | 3 | State specific objectives, including any prespecified hypotheses. | Introduction, final paragraph |
| Study design | 4 | Present key elements of study design early in the paper. | Methods 2.1 and 2.3 |
| Setting | 5 | Describe the setting, locations, and relevant dates, including periods of recruitment, exposure, and data collection. | Methods 2.1 |
| Participants | 6(a) | Give eligibility criteria and the sources and methods of selection of participants. | Methods 2.1; Supplementary Figure S1; Table S3 |
| Participants | 6(b) | For matched studies, give matching criteria and numbers of exposed and unexposed participants. | Not applicable; no matching was used |
| Variables | 7 | Clearly define outcomes, exposures, predictors, potential confounders, and effect modifiers; give diagnostic criteria if applicable. | Methods 2.2 |
| Data sources/measurement | 8 | For each variable, give data sources and details of assessment methods; describe comparability across groups or survey waves. | Methods 2.1–2.2; Supplementary Tables S1–S3 |
| Bias | 9 | Describe efforts to address potential sources of bias. | Methods 2.1 and 2.3; Results 3.1; Discussion, limitations; Table S3 |
| Study size | 10 | Explain how the study size was arrived at. | Methods 2.1: all eligible complete cases from the two selected public-use waves; no formal sample-size calculation |
| Quantitative variables | 11 | Explain how quantitative variables were handled; describe groupings and reasons for them. | Methods 2.2.1 and 2.2.4; Table S3, Panel D |
| Statistical methods | 12(a) | Describe all statistical methods, including those used to control for confounding. | Methods 2.3; Tables 2–3; Supplementary Tables S4–S8 |
| Statistical methods | 12(b) | Describe methods used to examine subgroups and interactions. | Methods 2.3; Results 3.3–3.7 |
| Statistical methods | 12(c) | Explain how missing data were addressed. | Methods 2.1 and 2.3; Results 3.1; Figure S1; Table S3 |
| Statistical methods | 12(d) | If applicable, describe analytical methods accounting for the sampling strategy. | Methods 2.3; Results 3.7; Supplementary Table S8 |
| Statistical methods | 12(e) | Describe any sensitivity analyses. | Methods 2.3; Results 3.6–3.7; Supplementary Tables S6–S8 |
| Participants/results | 13(a) | Report numbers of individuals at each stage of the study. | Results 3.1; Supplementary Figure S1; Table S3 |
| Participants/results | 13(b) | Give reasons for non-participation or exclusion at each stage. | Methods 2.1; Supplementary Figure S1; Table S3 |
| Participants/results | 13(c) | Consider use of a flow diagram. | Supplementary Figure S1 |
| Descriptive data | 14(a) | Give characteristics of study participants and information on exposures and potential confounders. | Table 1; Results 3.1 |
| Descriptive data | 14(b) | Indicate the number of participants with missing data for each variable of interest. | Supplementary Table S3, Panel B |
| Descriptive data | 14(c) | Summarize follow-up time when applicable. | Not applicable; pooled cross-sectional design |
| Outcome data | 15 | Report outcome events or summary measures. | Table 1; Results 3.1 and 3.3 |
| Main results | 16(a) | Give unadjusted and adjusted estimates and their precision; make clear which confounders were adjusted for and why. | Results 3.3–3.5; Tables 2–3; Supplementary Tables S4–S5 |
| Main results | 16(b) | Report category boundaries when continuous variables were categorized. | Methods 2.2.1 and 2.2.4; Table 1; Figure 1 |
| Main results | 16(c) | When relevant, translate estimates of relative risk into absolute risk for a meaningful time period. | Not applicable; continuous cross-sectional outcomes |
| Other analyses | 17 | Report other analyses, including subgroup, interaction, and sensitivity analyses. | Results 3.6–3.7; Supplementary Tables S6–S8 and Figure S2 |
| Key results | 18 | Summarize key results with reference to study objectives. | Discussion, opening paragraph |
| Limitations | 19 | Discuss study limitations, including direction and magnitude of potential bias or imprecision. | Discussion, limitations paragraph |
| Interpretation | 20 | Give a cautious overall interpretation considering objectives, limitations, multiplicity, and other evidence. | Discussion, paragraphs 2–7; Conclusion |
| Generalisability | 21 | Discuss the generalisability of the study results. | Discussion, strengths/limitations; Conclusion |
| Funding | 22 | Give the source of funding and the role of funders. | Funding declaration |

*Note. This checklist follows the STROBE reporting structure for cross-sectional studies. “Not applicable” is used where the item does not apply to the pooled cross-sectional design.*
